# MALDI-TOF imaging analysis of benzalkonium chloride penetration in *ex vivo* human skin

**DOI:** 10.1371/journal.pone.0297992

**Published:** 2024-02-08

**Authors:** Chase N. Morse, Collin C. Hite, Nathan C. Wamer, Jennifer N. Gadient, Gabriella Baki, Erin G. Prestwich

**Affiliations:** 1 Department of Medicinal and Biological Chemistry, College of Pharmacy and Pharmaceutical Sciences, The University of Toledo, Toledo, Ohio, United States of America; 2 Natural Sciences and Mathematics Instrumentation Center, The College of Natural Sciences and Mathematics, The University of Toledo, Toledo, Ohio, United States of America; 3 Department of Pharmacy Practice, College of Pharmacy and Pharmaceutical Sciences, The University of Toledo, Toledo, Ohio, United States of America; Alexandria University, EGYPT

## Abstract

Benzalkonium chloride (BZK), alkyldimethylbenzlamonium chloride, is a cationic surfactant that is used as an antiseptic. BZK is classified as a quaternary ammonium compound composed of molecules of several alkyl chains of differing lengths, that dictate its effectiveness towards different microbes. As a result, BZK has become one of the most used preservatives in antibacterial solutions. Despite its widespread use, it is not clear whether BZK penetrates human skin. To answer this question, BZK treated skin was analyzed using matrix assisted laser desorption ionization time-of-flight (MALDI-TOF) mass spectrometry imaging. Solutions containing BZK and differing excipients, including citric acid, caprylyl glycol, and vitamin E, were applied *ex vivo* to excised human skin using Franz diffusion cells. Treated skin was embedded in gelatin and sectioned prior to MALDI-TOF imaging. BZK penetrates through the epidermis and into the dermis, and the penetration depth was significantly altered by pH and additives in tested solutions.

## Introduction

Benzalkonium chloride (BZK) is a widely utilized class of quaternary ammonium molecules that can be used on a probationary basis in consumer antiseptic rub and wash products in the United States (US) [1]. Quaternary ammonium molecules function as lytic cationic biocides, which induce cell lysis and disrupt membranes [2]. They can also enhance penetration of other compounds by disrupting the lipid packing in the stratum corneum of the skin [3]. Due to this, BZK is used as an antiseptic in consumer rubs and washes for topical solutions [4]. BZK contains the structure [C_6_H_5_CH_2_N(CH_3_)_2_R]Cl where R is a mixture of alkyl chains: *n*-C_8_H_17_, *n*-C_10_H_21_, *n*-C_12_H_25_, *n*-C_14_H_29_, and *n*-C_16_H_33_ [5, 6]. The length of the carbon chain is important in determining whether BZK will kill specific classes of microbes. The two main forms of BZK are benzyldimethyldodecylammonium chloride (C_12_), *m/z* 304.30, that can be effective against fungi, and benzyldimethyltetradecylammonium chloride (C_14_), *m/z* 332.33, that can be effective against Gram-positive bacteria and some Gram-negative bacteria [5, 7, 8]. Currently, in any BZK antiseptic solution marketed in the US, the FDA stipulates that the concentration of BZK should be between 0.1% and 0.13% [1]. Additionally, the US Pharmacopeia (USP) requires specific alkyl lengths: C_14_ must be 40% of the solution while C_12_ must compose at least 20% [9]. The USP further requires the C_12_ and C_14_ alkyl lengths must be 70% of the BZK molecules in a solution. Benzyldimethylhexadecylammonium chloride (C_16_), *m/z* 360.36, which is less regulated and sometimes present, has been shown to be effective against differing classes of bacteria [5, 7]. Despite extensive application, concerns remain regarding the overall safety of BZK. Studies have shown that several routes of administration at high concentrations of BZK can become toxic [10–12]. Additionally, BZK can trigger allergic reactions causing conjunctivitis and contact dermatitis following application [13]. Because BZK is common in ocular treatments, previous studies examined the localization of BZK in the eye [14, 15]. However, the penetration of BZK into the layers of human skin has not been determined.

Matrix assisted laser desorption ionization time-of-flight (MALDI-TOF) mass spectrometry imaging is an analytical technique, which allows for rapid label free analysis of large analyte regions [16]. This method provides insight into ion localization within selected regions depicted by ion heat maps. The technique has been employed in pharmaceutical research to monitor drug and metabolite distribution within organ and tissue samples [16–20]. Typically, this is done by mounting samples to an indium titanium oxide (ITO) slide after they have been sectioned. Samples can be sectioned and adhered either with or without the use of an embedding media [17, 18, 21–24]. These embedding media are used to provide additional support and greater control over sample handling and orientation. However, many commonly utilized embedding media contain polymeric compounds that can ionize in the MALDI and interfere with the analytes of interest [22]. Therefore, alternative embedding methods have been explored that lack background ion interferences. Usage of gelatin-solutions as embedding media have emerged as a promising option since gelatin has little to no background interference and has little effect on sample structure or shape [22].

Human skin is composed of three layers, the outermost is the epidermis followed by the dermis, and finally the subcutaneous membrane [25, 26]. The epidermis is further divided into four or five sublayers, depending on the body site. The stratum corneum, the outermost epidermal layer, functions as the primary skin barrier to hold in moisture while preventing foreign substances from entering [3, 25]. The basement membrane is a porous intersection between the epidermis and dermis that regulates the exchange of molecules. The dermis, the largest layer, is composed of connective tissue and contains fibroblasts, hair follicles, sensory organs, nerves, sweat glands, oil glands, and blood vessels [25]. Both the epidermis and dermis are influenced by factors such as skin region, sex, age, skin tone, weight, and seasons of the year, resulting in a large amount of variability in the exact skin layer widths [27–31]. Finally, the subcutaneous membrane is the layer of fat between the dermis and the muscles, major vasculature, and other internal organs [25]. To analyze the skin layers using MALDI-TOF imaging, the skin must be perpendicularly sectioned. Since skin is flexible, additional support may be required to section the skin uniformly. Embedding techniques, such as gelatin, enable consistent sample preparation and analysis of ion localization within the layers of human skin.

In this study, different solutions containing 0.123% (w/v) BZK were applied to human skin *ex vivo* and analyzed to determine the extent of skin BZK penetration. Franz diffusion cells are used to study drug penetration on *ex vivo* human skin [17, 32]. Franz diffusion cells contain two separate pieces, a top donor chamber and a bottom receptor chamber. Skin pieces are placed and clamped between the chambers to allow application of test solutions [32]. To understand if common excipients found in hand sanitizers changed the ability of BZK to enter skin, citric acid, caprylyl glycol, and vitamin E were added to BZK and tested. Each of these excipients in cosmetic solutions are used as preservatives, emulsion agents, and antioxidants [33]. Previous studies have shown that caprylyl glycol [34] and vitamin E [35, 36] can influence the penetration of drugs. Two commercial solutions which contain a mixture of several excipients were also analyzed. Treated skin samples were embedded in gelatin, sectioned, and analyzed using MALDI imaging to map the BZK ions, *m/z* 304 and *m/z* 332, within the tissue.

## Materials and methods

### Materials

PolyFreeze tissue freezing medium, 10x phosphate-buffered saline (PBS), α-cyano-4-hydroxycinnamic acid (α-CHCA), α-(+)-tocopherol acetate (Vitamin E), 1,2-octanediol (caprylyl glycol), acetic acid, hematoxylin, and eosin Y were purchased from Sigma Aldrich (St. Louis, MO). Citric acid monohydrate was purchased from Honeywell (Morris Plane, NJ). Benzalkonium chloride (BZK) part #12060 was purchased from MilliporeSigma (Burlington, MA). Ethanol (190 proof) part #2801 was purchased from Deacon Labs Inc. (King of Prussia, PA). Kroger brand 100% pure vegetable oil was purchased from Kroger (Cincinnati, OH). Great Value Brand unflavored gelatin was purchased from Wal-Mart (Bentonville, AK). Indium titanium oxide (ITO) slides were purchased from Bruker (Billerica, MA). Double sided copper tape was purchased from Electron Microscopy Sciences (Hatfield, PA). Microtome blades and specimen mounting discs were purchased from Leica Biosystems (Wetzlar, Germany). Antimicrobial solutions 1 and 2 were provided by Rockline Industries (Sheboygan, WI). Ultrapure water was made from Milli-Q water using a Millipore Reference system (Millipore, Burlington, MA).

Flash frozen human skin were purchased from Zen-Bio (Durhman, NC). A total of three skin tissue samples (catalog #T-SKN-FF10CM) were purchased, which included arm skin from a 44-year-old African American female, thigh skin from a 41-year-old Caucasian female, and abdominal skin from a 32-year-old Caucasian female. All were non-diabetic and non-smoker and removed during elective surgeries. Skin samples were received de-fatted and stored at -20°C.

### Solution preparation

Seven solutions were analyzed in this study; all solutions were made from ultrapure water. The positive control consisted of 0.123% (w/v) BZK in water. The negative control consisted of only ultrapure water. Two commercial solutions containing 0.123% BZK noted as either Solution 1 or Solution 2, were used with contents of each listed in S1 Table. The BZK + citric acid solution was composed of 0.123% (w/v) BZK in water and pH adjusted to 4.7 with citric acid. The BZK + vitamin E solution was comprised of 0.001% (v/v) vitamin E prepared in ethanol and then added to a solution 0.123% (w/v) BZK in water and then was pH adjusted to 4.7 with citric acid. A BZK + caprylyl glycol solution was prepared with 1.0% (w/w) caprylyl glycol and added to 0.123% (w/v) BZK in water and then adjusted the pH to 4.7 with citric acid. The alkyl chain distributions within utilized BZK solutions are listed in S2 Table.

### Skin treatment

*Ex vivo* human skin dermatomed to full thickness was thawed in room temperature 10x phosphate-buffered saline (PBS). The receptor chamber was filled with 12 mL of 10X PBS (pH 7.4), held under constant stirring with a magnetic stir bar at 300 rpm, and temperature maintained at 32 ± 0.5°C. Large pieces of skin were cut into smaller pieces to an appropriate size to allow a diffusion area of 1.77 cm^2^ and were clamped between the donor and receptor chambers of the Franz diffusion cells and equilibrated for 45–50 minutes. Any air bubbles formed in the receptor chamber were removed through the side arm of the cell. Following equilibration, 150 μL of the test solution was pipetted onto each skin sample such that the liquid spread across the skin. After 30 seconds, the liquid solutions were removed from the skin using two sterile cotton swabs. The skin remained clamped for one hour before being removed with tweezers and stored at -80°C until sectioning. Four pieces of skin were treated separately with the BZK + citric acid, BZK + vitamin E, and BZK + caprylyl glycol solutions. Six pieces of skin were treated with the BZK positive control, the water negative control, the solution 1, and the solution 2 solutions.

### Embedding medium for skin

Treated skin pieces were cut in half using a scalpel from epidermis to dermis for embedding. The skin pieces were embedded to allow the epidermis and dermis to be visible. Two embedding methods were tested in this study. The first method utilized PolyFreeze tissue freezing medium. The skin piece samples were placed in a small weigh boat and filled with PolyFreeze medium. The skin tissue in freezing media was solidified at -20°C, and then removed from the weight boat. The second method utilized gelatin. Sterile 6-well culture plates were filled with ultrapure water and 15% (w/v) unflavored gelatin was added and mixed at room temperature (24°C) [22]. Skin samples were placed in the mixture and stored uncovered for one hour at 4°C to fully solidify the gelatin. Excess gelatin around the skin was cut away and removed. Encased samples were stored at -20°C until sectioning.

### Skin sectioning and mounting

Samples were sectioned using a Leica CM1950 clinical cryostat (Leica, Wetzlar, Germany). Both PolyFreeze and gelatin embedded samples were transported on dry ice. Samples were adhered to 30 mm specimen object discs using PolyFreeze tissue freezing medium and placed into cryostat with chamber temperature set to -14°C. The PolyFreeze did not touch the tissue when gelatin embedding was used. Once fully solidified the specimen object discs were attached to the specimen head set to -20°C. Tissues were sectioned with Leica 818 High Profile Microtome Blades with a trim setting of 50 μm and a section setting of 20 μm. The blade sectioned the skin perpendicularly, allowing the epidermal and dermal layers to be cut simultaneously. A minimum of three sections were sectioned for each treatment group which were thaw mounted onto indium titanium oxide (ITO) (Bruker, Billerica, MA) slides with embedding media still present. No additional skin washing was used. The sample slides were then stored at -20°C until sublimation. All parts of the cryostat including the specimen object discs were cleaned with 200 proof ethanol and blades were replaced between each of the treatment groups.

### Sublimation

A mixture of 200 mg of α-CHCA and 5 mL of acetone was placed in the bottom of a sublimation apparatus. The mixture was used to coat the bottom of the sublimation apparatus and dried with a stream of air. The ITO slides were fixed to the top piece of the sublimation apparatus with double sided copper tape and placed under vacuum. Heat was applied to reach a temperature of 175–200°C and α-CHCA was sublimed for 20–25 minutes. Teaching points were added with a metallic marker and calibrant was added following sublimation.

### MALDI-TOF

Samples were analyzed with a Bruker UltrafleXtreme MALDI-TOF/TOF and flexControl software. This instrument was tested at a resolution of ≥ 13,000 m/dm for masses below 900 Da. Both scans and MALDI imaging were conducted in reflectron positive mode with a frequency-tripled Nd:YAG laser (355 nm). For scans, a laser intensity of 10% to 35% at an m/z range of 100–600 was used, and the laser was set to partial sample walk using 5,000 laser pulses. The instrument was calibrated to less than 5 ppm using elemental sulfur cluster, S_11_ (*m/z* 351.693), prior to collecting all scans and images [37]. To confirm the presence of the ions of interest, prepared solutions were spotted with 40 mg/mL CHCA mixed with acetone. Collision induced dissociation (CID) with nitrogen gas was performed on the ions *m/z* 304.3 and *m/z* 332.33 present in the 0.123% BZK solution.

MALDI imaging covered a mass range of 110 to 610 Da with a laser intensity of 60%, raster width of 200 μm, and both fuzzy controls and matrix suppression set to off. Fifty laser shots were collected for each raster spot. Teaching points were added with a metallic marker to locate regions of interest which were based on the size and shape of mounted skin. A pixel size of 50 μm was used for all MALDI imaging experiments. Total ion count (TIC) normalization was used in the Bruker flexImaging software.

### Hematoxylin & eosin Y (H & E) staining

Following MALDI imaging, slides were stored at -20°C until staining. Slides were first exposed to an ethanol dehydration gradient. Samples were stained in the following manner first with a Mayer’s hematoxylin solution followed by a water wash. Next, a 0.2% (v/v) ammonia solution was used as a bluing solution followed by an ethanol wash. Finally, tissues were counter stained with eosin Y and washed with ultrapure water and ethanol. Vegetable oil was used to remove any residual stain [38–40]. Stained slides were viewed, and pictures taken on an Olympus SZX7 light microscope with WHSZ 10X eyepieces and an SC100 camera attached (Olympus, Shinjuku City, Tokyo, Japan). A magnification of 1.6X and pixel size of 2 μm was utilized for all measurements.

### Data analysis

The H & E stained slides were analyzed using Olympus cellSens software to determine the skin epidermal thicknesses. Measurements of the epidermis thickness were taken perpendicular from the skin surface to the basement membrane. The number of measurements taken varied based on the size of the sectioned skin. For BZK penetration measurements, ion heat maps for *m/z* 304.3 ± 0.76 and *m/z* 332.33 ± 0.83 were exported from flexImaging to ImageJ. Twenty ion measurement points distributed across the length of the skin were taken in a straight line from the surface of the skin to the ion at the deepest point to give total ion penetration. For proper measurements, the scale bar from flexImaging was measured in ImageJ to determine their pixel length. This pixel length for each exported image was converted into its corresponding micrometer length. If no ion signal was present at a measurement point, it was recorded as 0 μm of penetration. The ion penetration into the dermis of each sample was determined by subtracting the average epidermal width, from H & E staining of the specific treatment group, from the calculated total ion penetration of the particular sample. Co-localization analysis was calculated using the JaCoP plugin of ImageJ [41]. A Pearson’s Correlation Coefficient x > 0.7 was considered to be colocalized [41].

Statistical analysis was conducted with GraphPad Prism (Version 9.4.0). Significance between treatment groups ion penetration and epidermis thicknesses were calculated using one-way ANOVA with *post hoc* Tukey’s test for correction of multiple comparisons. Student’s paired t-test were performed to determine significance within treatment groups ion penetrations and epidermis thicknesses. Results were considered significant at P < 0.05 * or lower (P<0.01 **, P< 0.001 ***, P<0.0001 ****).

### Ethics statement

All flash frozen human skin was purchased directly from Zen-Bio (Durham, NC) who obtained skin from anonymized, consenting, adult volunteers undergoing elective surgeries. Signed Institutional Review Board (IRB) validated donor consent forms were collected by Zen-Bio stating skin would be utilized for non-clinical research. All preprocessing of skin utilized by Zen-Bio followed Standard Operating Procedure managed GLP protocols compliant with ethical regulations.

## Results

### Matrix selection and analysis of solution compositions

MALDI scans were taken of matrices as well as test solutions and their components (S1 Fig). Due to the lack of interfering ions, α-CHCA was selected for the analysis (S1A Fig). Initially, the matrix mixture of 2,5-dihydroxybenzoic acid:2-hydroxy-5-methoxybenzoic acid (9:1) (sDHB) was used; however, an interfering ion *m/z* 332.14 was found (S1B Fig). Scans of 0.123% BZK clearly demonstrated the *m/z* 304 and *m/z* 332 ions that correspond to the C_12_ and C_14_ chain lengths (S1C Fig; S3 Table). Both ions were further fragmented to confirm their structures by CID (S2 Fig). Of note, the ion *m/z* 360 which corresponds to the C_16_ BZK chain length is visible in scans (S1C Fig); however, the ion *m/z* 360 failed to appear using MALDI imaging due to a low signal to noise ratio. Furthermore, FDA guidelines are less restricted on the usage of C_16_ BZK, and it was not further analyzed. The spectra of the negative water control and the individual excipients (citric acid, vitamin E, and caprylyl glycol) did not include the BZK *m/z* 304 and *m/z* 332 ions (S1D–S1F Fig). Scans for treatment solutions that included BZK displayed clear presence of the *m/z* 304 and *m/z* 332 ions (S1C, S1G–S1K Fig).

### Sample embedding

To determine average penetration depth of BZK in skin, ions m/z 304 and m/z 332 were analyzed by MALDI imaging. To do this, samples were treated, embedded, and sectioned to view both the epidermal and the dermal skin layers. In our hands, we could not properly section the skin and adhere the section to the ITO slides without an embedding media or embedding with water. The skin was embedded in PolyFreeze tissue freezing medium. However, upon MALDI imaging, ions m/z 304 and m/z 332 were observed in the negative test samples (S3A–S3E Fig). To address these interfering ions, gelatin was used as an embedding medium. Skin slices were easily suspended in gelatin and negative controls lacked the presence of the ions m/z 304 and m/z 332 (S3F–S3J, S4A–S4C Figs). For positive controls both the m/z 304 and m/z 332 ions appeared at a high intensity in the epidermis (S4D and S4E Fig) with lower intensities observed deeper into the dermis (S4F and S4G Fig). Gelatin alone without skin did not contain either the m/z 304 or m/z 332 ions (S5 Fig). Neither the negative nor the positive controls contained ion peaks of the epidermis and dermis which overlapped with the BZK ions (S4 Fig). Gelatin was tested at different percentages, including 10%, 15%, 20%, and 25% to balance the level of gel hardness and the time required for gel solidification. Higher concentrations (i.e., 20 and 25%) solidified immediately and did not allow the skin to be positioned for slicing. The lowest concentration (10%) was brittle after freezing, and the samples flaked off and broke when sectioned. The chosen 15% gelatin solution allowed enough time to properly suspend the skin and permit even cutting.

Samples embedded in gelatin were tested to ensure they could withstand the MALDI imaging process. Before MALDI imaging, the matrix must be sublimed onto the samples. Following sublimation, skin was imaged. The gelatin mounted skin remained adhered to the slide through the entire process. After MALDI imaging, the tissues were stained, and the skin typically remained on the slide. The skin detached from the slides in only a few cases. Overall, using the gelatin-based embedding medium was effective for the analyses, and thus it was used in this study.

### Epidermis thickness

To analyze the potential of BZK to penetrate the dermis, the thickness of the epidermis was determined. The epidermal thickness of each sample was measured from the top of the skin to the basement membrane using straight lines [42]. The number of measurements taken of the epidermis was based on the size of the skin section, but an average of 32 measurements per section were taken. Initially, epidermal thicknesses were analyzed as separate treatment groups based on the specific skin location: either arm, thigh, or abdomen (S6A Fig; S4 Table). When comparing the epidermal thicknesses within a treatment group across the specific skin locations, the differences were mostly non-significant. The lone exception was the BZK + caprylyl glycol solution, which showed a significant difference in observed thickness between the thigh and abdomen (p-value 0.0118) (S6A Fig). Since there were minimal differences between the skin locations and our observed epidermal widths were similar to those observed in other studies [27–31]; the skin locations were combined for further analyses (Table 1; S6B Fig).

**Table 1:** Determined thickness of the epidermis of each treatment group regardless of the skin origin.

	Epidermis width (μm)		
	Average	SD	N
BZK in MilliQ water	92	7	4
MilliQ water	81	2	8
BZK + citric acid (pH 4.7)	83	9	4
Solution 1	69	4	21
Solution 2	82	6	13
BZK + caprylyl glycol	79	8	9
BZK + vitamin E	95	5	9

All measurements were taken from the surface of the skin to the basement membrane and were done on stained skin following MALDI imaging. Measurements were recorded in μm and N is the total number of skin sections successfully stained and analyzed.

The combination of all skin locations revealed significant differences between the epidermal thickness based on the treatment applied (S6B Fig). The BZK + vitamin E solution had the thickest epidermis with an average thickness of 95 ± 5 μm, which was significantly higher than skin treated with only water (negative control) 81 ± 2 μm (S6B Fig). The positive control showed a significant increase in epidermal thickness of 92 ± 7 μm compared to the negative control (Table 1; S6B Fig). The thinnest epidermis was observed with Solution 1 (69 ± 4 μm), which was a significant decrease from the positive and negative control groups as well as the BZK + vitamin E solution (Table 1; S6B Fig). Solution 1 also contained caprylyl glycol but was significantly thinner than skin treated with BZK + caprylyl glycol, which had an average epidermis thickness of 79 ± 8 μm (Table 1; S6B Fig). Solution 2 had an average thickness of 82 ± 7 μm, which was slightly higher than the negative control, but significantly lower than the positive control (Table 1; S6B Fig).

Control experiments were conducted to determine if the epidermis was altered in each step of the analysis. Select samples were stained following mounting, sublimation, or imaging (S7A Fig). The skin was thinner after mounting than after MALDI imaging and staining (S7B Fig). This increase could be due to damage and dehydration occurring to the epidermis causing it to flatten and spread-out following sublimation or imaging. Based on these control experiments, the noted epidermal thicknesses may be slightly higher than when the skin was initially treated. However, the penetration depths of BZK into the skin were still found to significantly differ between the treatment groups (S8 Fig).

### Penetration depths of BZK ions *m/z* 304 and *m/z* 332

**Fig 1:**
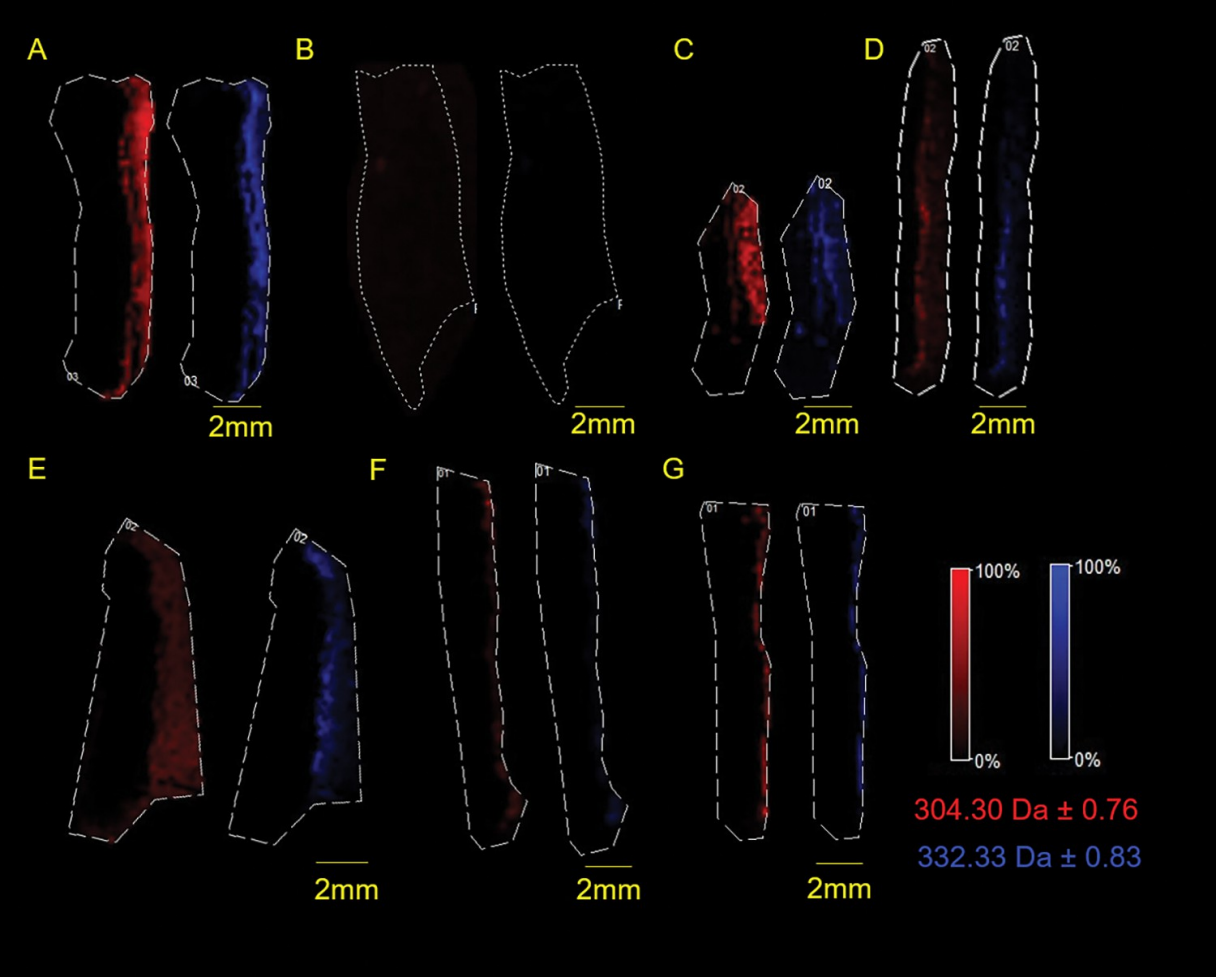
Treated skin single ion heat maps. Selected ion heat maps of **(A)** BZK in water, **(B)** water, **(C)** Solution 1, **(D)** Solution 2, **(E)** BZK + citric acid (pH 4.7), **(F)** BZK + caprylyl glycol, **(G)** BZK + vitamin E. The ion *m/z* 304.30 is shown in red and ion *m/z* 332.33 in blue. All yellow scale bars represent 2 mm. Skin is oriented with epidermis on the right side. Dotted lines are the selected regions of interest drawn along the edges of the skin.

To determine the penetration of BZK into the skin samples, the MALDI imaging ion heat maps of ions *m/z* 304 and *m/z* 332 were analyzed. Both ions were normalized using total ion count (TIC) before being exported to ImageJ. The distance from the surface of the skin to the furthest point of observed ion intensity was measured. Representative ion heat maps of *m/z* 304 ion and *m/z* 332 ion for each treatment group are shown in Fig 1. Penetration depths into the skin (epidermis and dermis together) were determined for both ions *m/z* 304 and *m/z* 332 which showed significant differences in BZK penetration between the treatment groups (S8 Fig).

**Fig 2:**
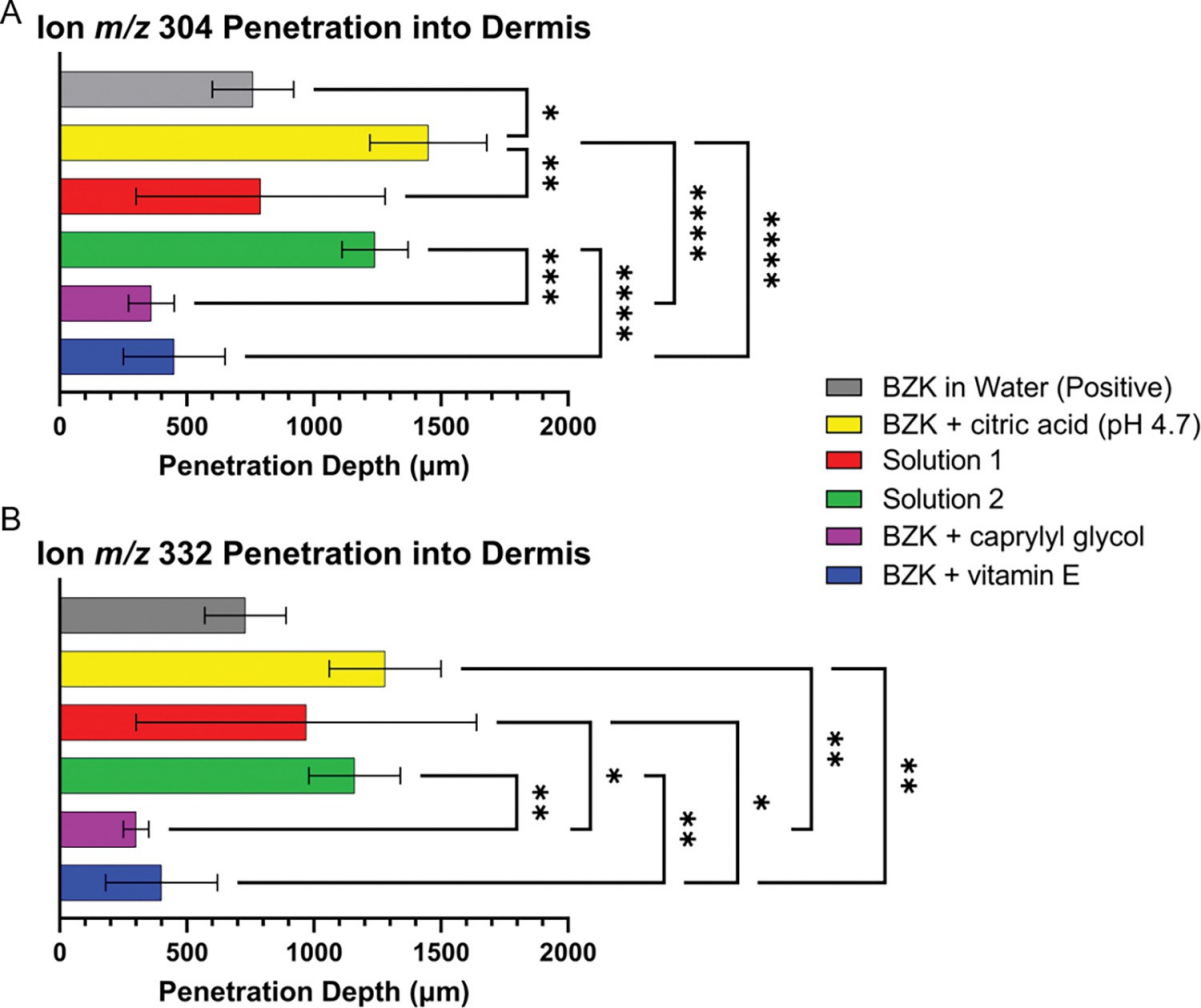
Graph of depths of single BZK ions into dermis layer of skin. **(A)** Bar chart of average depth of ion *m/z* 304 into the dermal layer. **(B)** Bar chart of average depth of ion *m/z* 332 into the dermal layer. One-way ANOVA was performed to determine significance where P<0.05 *, P<0.01 **, P< 0.001 ***, and P<0.0001 ****.

To better understand BZK penetration into the dermis, the average thickness of the epidermis was subtracted from each individual skin section that was imaged. When the epidermis was subtracted from each group, it was clear that BZK penetrated differently depending on pH and excipients. Ion depths were measured for both the *m/z* 304 and *m/z* 332 ions (Fig 2; Table 2). The BZK + citric acid solution penetrated the deepest with the *m/z* 304 ion reaching an average of 1450 μm and the *m/z* 332 ion reaching 1280 μm into the dermis (Table 2). The ion *m/z* 304 penetrated significantly deeper than the positive control group (BZK in water), which only reached 760 μm (Fig 2A). Ion m/z 332 did not differ significantly from the positive control, which averaged a penetration depth of 730 μm (Fig 2B). The BZK + caprylyl glycol solution had the least observed penetration of 360 μm for the *m/z* 304 ion and 300 μm for the *m/z* 332 ion (Table 2). The BZK + vitamin E solution showed depths of 450 μm and 400 μm for the *m/z* 304 ion and the *m/z* 332 ion, respectively (Fig 2; Table 2). Neither the BZK + caprylyl glycol solution, nor the BZK + vitamin E solution were found to be significantly different from the positive control or from one another (Fig 2). However, both solutions had significant decreases in BZK penetration when compared to the BZK + citric acid solution (Fig 2).

**Table 2:** Observed average depth of BZK into the dermal layer of skin.

	Ion *m/z* 304 penetration into dermis		
	Average	SD	N
BZK in MilliQ water	760	160	3
BZK + citric acid (pH 4.7)	1450	230	4
Solution 1	790	490	10
Solution 2	1240	130	6
BZK + caprylyl glycol	360	90	6
BZK + vitamin E	450	200	11
	Ion *m/z* 332 penetration into dermis		
	Average	SD	N
BZK in MilliQ water	730	160	3
BZK +citric acid (pH 4.7)	1280	220	4
Solution 1	970	670	10
Solution 2	1160	180	6
BZK + caprylyl glycol	300	50	6
BZK + vitamin E	400	220	11

Measurement values in μm for both ion *m/z* 304 and ion *m/z* 332 are listed for each of the treatment groups. Where N is the number of skin sections analyzed with MALDI imaging.

Commercial BZK solutions (Solutions 1 and 2) were analyzed using the same methods. These solutions contained the same BZK percentage but differed in their excipients (S1 Table) and pH (Solution 1 at 4.7 and Solution 2 at pH 3.1). The BZK in Solution 1 penetrated to 790 μm and 970 μm for ions *m/z* 304 and *m/z* 332, respectively (Table 2). Solution 2 penetrated ion depths of 1240 μm and 1160 μm (Table 2). Neither were found to differ significantly from the positive control (Fig 2). However, the *m/z* 304 ion penetrated significantly less when Solution 1 was tested as compared to the BZK + citric acid solution (Fig 2A). Although both Solutions 1 and 2 contained largely differing mixtures of excipients, both contained vitamin E. When comparing the commercial solutions to the BZK + vitamin E solution, Solution 2 was significantly different for both the *m/z* 304 ion and the *m/z* 332 ion (Fig 2), and in Solution 1 the *m/z* 332 ion penetrated significantly deeper (Fig 2B). Caprylyl glycol was only present in Solution 1 and when compared to the BZK + caprylyl glycol solution, a significant increase in penetration was observed only for the *m/z* 332 ion (Fig 2B). The *m/z* 304 ion penetrated deeper in Solution 1 but was nonsignificant when compared to the BZK + caprylyl glycol solution (Fig 2A).

### Differences in BZK ion penetration

**Fig 3:**
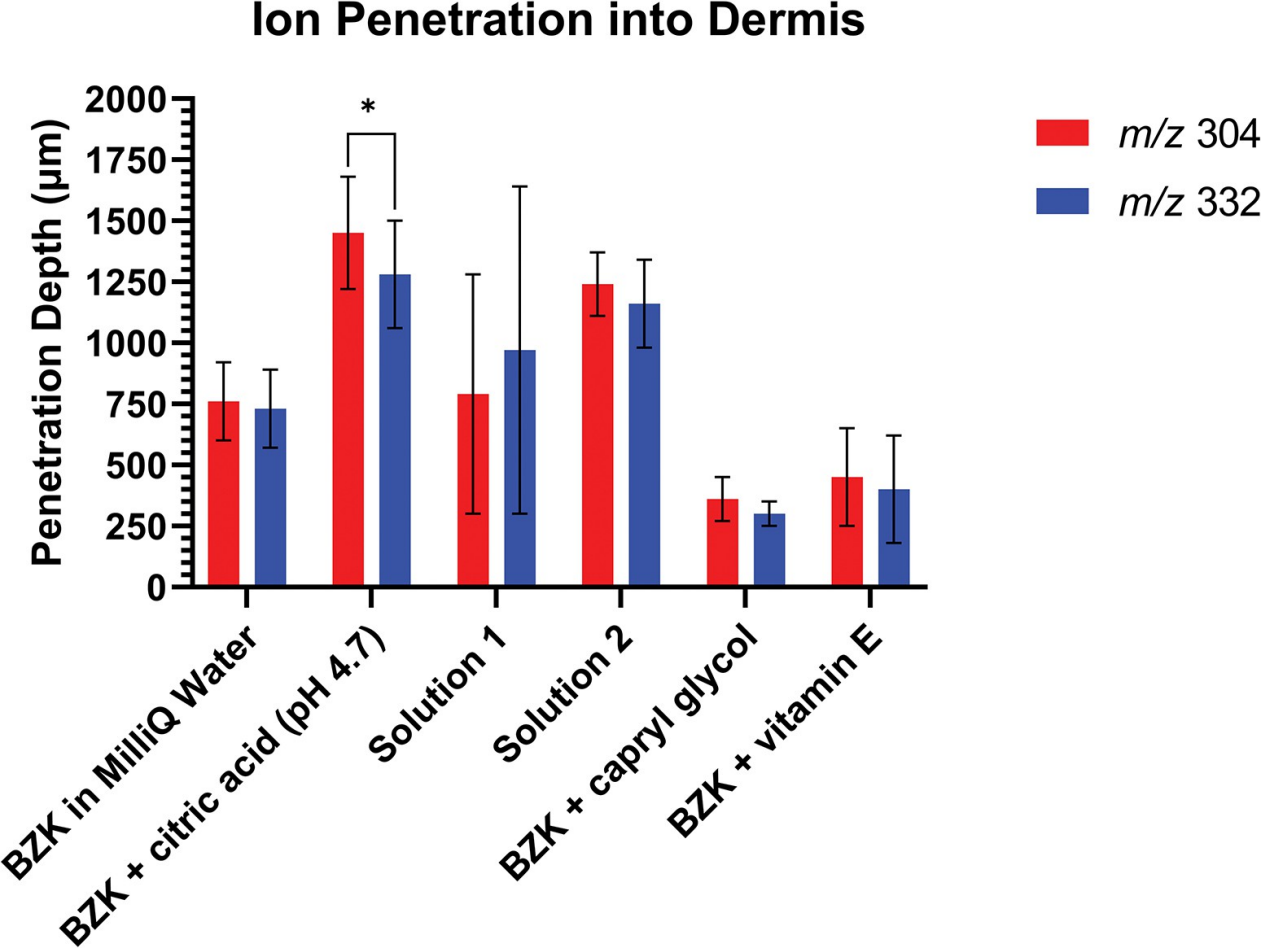
Ion *m/z* 304 to ion *m/z* 332 comparison. Comparisons of the observed average depths of ions *m/z* 304 and *m/z* 332 into the dermis. Paired t-tests were performed to determine significance where P<0.05 *, P<0.01 **, P< 0.001 ***, and P<0.0001 ****.

Solutions were analyzed to determine if the two BZK ions in a treatment group entered different human skin layers. It appeared that the *m/z* 304 ion could have penetrated deeper into the skin (Fig 3, S5 Table) but only the BZK + citric acid solution differed significantly (p-value of 0.0250) between the *m/z* 304 ion and the *m/z* 332 ion (Fig 3).

A co-localization analysis was performed to determine if the ions *m/z* 304 and *m/z* 332 ionize in the same location in the tissue. Most samples showed co-localization of the BZK ions with Pearson’s correlation coefficients greater than 0.7 (S6 Table). However, the BZK + citric acid solution failed to show co-localization for two of the samples. Additionally, the BZK + citric acid solution significantly differed in the depths of the *m/z* 304 ion compared to the *m/z* 332 ion (Fig 3). For the other solutions, three of the eleven of the BZK + vitamin E samples, two of the ten Solution 1 samples, and two of the six Solution 2 samples did not have a Pearson’s value ≥ 0.7 (S6 Table). However, for all these samples, the average correlation coefficient of the treatment groups was greater than 0.7. The BZK + citric acid solution was the only group which had an average correlation coefficient below the 0.7 threshold.

### BZK ion penetration of the skin

**Fig 4:**
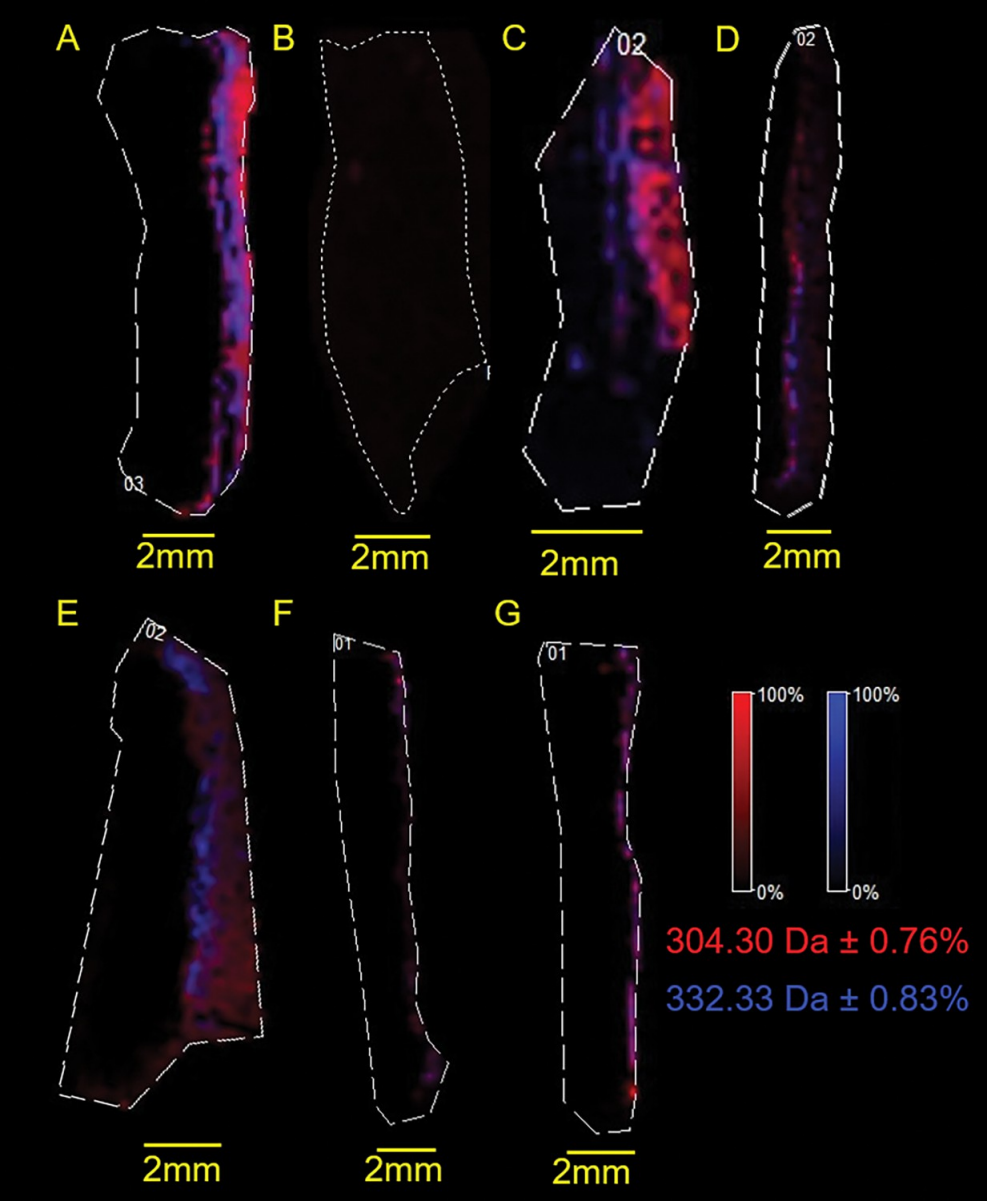
General penetration of BZK ion heat maps. Selected ion heat maps of **(A)** BZK in water, **(B)** water, **(C)** Solution 1, **(D)** Solution 2, **(E)** BZK + citric acid (pH 4.7), **(F)** BZK + caprylyl glycol, **(G)** BZK + vitamin E. The ion *m/z* 304.30 is shown in red and ion *m/z* 332.33 in blue. All yellow scale bars represent 2 mm. Skin is oriented with epidermis on the right side. Dotted lines are the selected regions of interest drawn along the edge of the skin.

**Fig 5:**
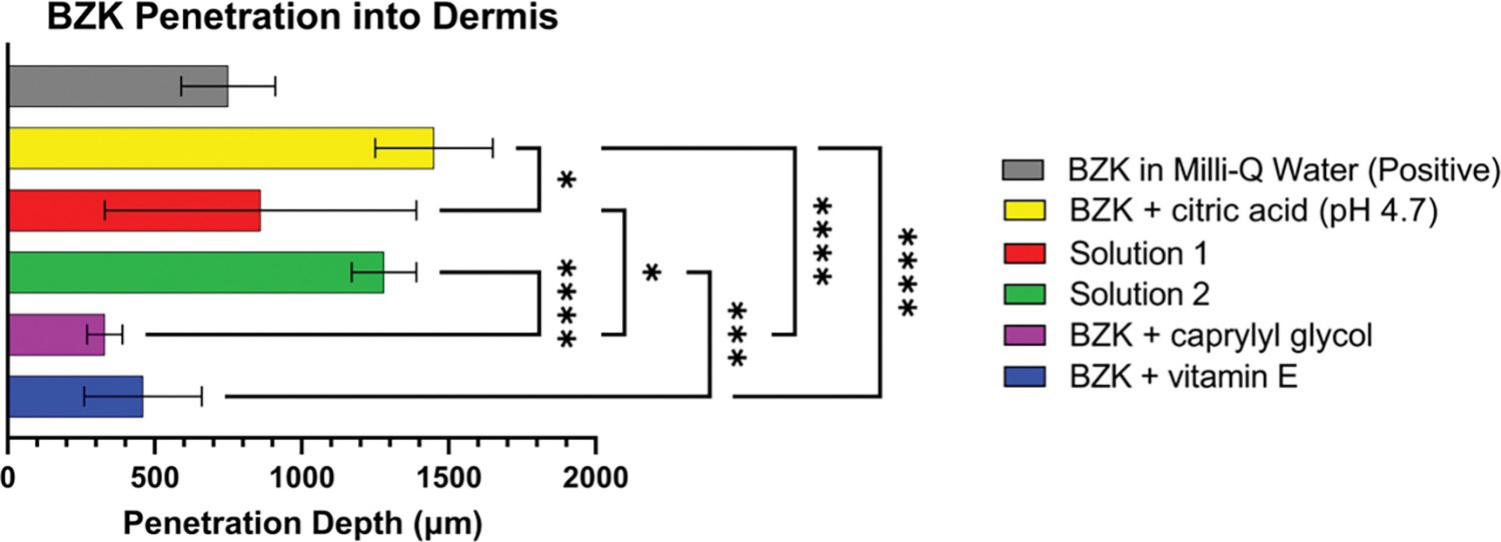
General BZK penetration into dermis. Graph showing the determined BZK ion penetration based on ion heat maps showing both ion *m/z* 304 and ion *m/z* 332. One-way ANOVA was carried out to determine significance where P<0.05 *, P<0.01 **, P< 0.001 ***, and P<0.0001 ****.

The general depth of BZK was also analyzed by measuring ion heat maps of both the *m/z* 304 and *m/z* 332 ions (Fig 4). Though these ions co-localized for most samples, slight differences in ion depths and distribution were observed in the ion heat maps. Analyzing both ions on the same heat map revealed increased penetration into the dermis as compared to either the *m/z* 304 ion or the *m/z* 332 ion alone (Figs 4 and 5). This occurs because either ion can be counted if it penetrates deeper into the skin. Similar to the results observed for the individual ions, both ions penetrates significantly when tested with BZK + citric acid solution as compared to the BZK + vitamin E solution or the BZK + caprylyl glycol solution (Fig 5). The BZK ions significantly penetrated the skin in Solution 2 compared to the BZK + vitamin E solution. In Solution 1, BZK ions significantly penetrated the skin as compared to the BZK + caprylyl glycol solution (Fig 5).

## Discussion

Despite the widespread use of BZK in topical antiseptic solutions, there are questions about BZK skin safety. Therefore, it is important to determine whether BZK enters different layers of skin. The overall aim of this analysis was to first understand if BZK was capable of penetrating human skin and then to measure the extent of penetration.

### Treatment of skin

The skin was treated with BZK-containing solutions to study the penetration depths of BZK. Each solution contained the same w/v percentage of BZK (0.123%) and are within the FDA guidelines for BZK use in antimicrobial solutions [1]. The C_12_ and C_14_ BZK chain lengths were analyzed to focus on commercial solutions as suggested by the FDA. Solutions with specific excipients were made and used to determine if additives change BZK skin penetration. In general, the concentrations of ingredients were tested to allow solutions to be compared easily. The solutions that contained vitamin E all had the same concentration (BZK + vitamin E, Solution 1, and Solution 2). However, the amount of caprylyl glycol in the BZK + caprylyl glycol solution was higher (1.0% w/v) than Solution 1 (0.25% w/v). Citric acid was used to adjust the pH of the solutions made in our lab to pH 4.7, to match the pH of Solution 1. However, Solution 2 was pH 3.1 and was not adjusted further (S1 Table). To treat skin, samples were clamped into Franz diffusion cells. After equilibration, each solution was pipetted directly onto different skin pieces and were incubated for thirty seconds. The solutions were wiped off the skin and incubated in the Franz diffusion cells for an hour. Other studies analyzing molecule penetration into human skin using Franz diffusion cells used treatment windows of minutes to hours [17, 19]. The treatment parameters utilized for this study were selected to mimic a typical hand cleaning procedure where a small amount of a commercial solution containing BZK was applied to the skin and then quickly wiped away. Since the focus was to understand whether BZK penetrates skin and a consistent short application time was employed, the receptor fluid and the solution that was removed from the skin were not analyzed.

### Gelatin embedding

Establishing skin embedding parameters were initially challenging. First, PolyFreeze tissue freeze medium was used due to its popularity in imaging analyses; however, it was ineffective due to the presence of interfering ions (S3A–S3E Fig). Other groups have found that embedding media can contain contaminating ions in MALDI imaging [22]. Water has been used previously as an embedding medium; however, this medium can damage samples and water frozen within samples can displace ions of interest and distort MALDI images [43]. In our hands, neither skin prepared without an embedding media nor embedded with water properly adhered to the ITO glass slides. Gelatin based embedding mediums have been commonly used [22]. Skin can be specifically positioned in gelatin, and gelatin is sturdy but easy to cut. The gelatin did not alter the integrity of the skin or contain any contaminating ions (S5 Fig). The skin sections in gelatin readily adhered to the ITO glass slides (S3F–S3H, S4 and S5 Figs). However, samples in gelatin can disintegrate after three months in the freezer. Other freezing mediums contain polymers and preservatives to prevent deterioration of samples. Without these components, gelatin samples are more susceptible to dehydration and the formation of ice artifacts, which can affect slicing. With prolonged storage (>3 months), skin embedded in gelatin became brittle, causing challenges with proper slicing and adherence to the ITO slides. However, for samples prepared and used within three months of initial embedding the method proved effective.

When mounting, it was common for some of the gelatin to become loose from the ITO slides, but, the skin sections adhered throughout the imaging process. However, during staining, sometimes the skin sections detached from the glass slides. Overall, gelatin embedding medium proved to be effective for slicing, mounting, and imaging of skin samples in our study.

### Skin staining and epidermal thickness

Epidermal thicknesses were originally analyzed as distinct treatment groups depending on which body part the piece of skin was from: arm, thigh, or abdomen (S6A Fig; S4 Table). However, epidermal thicknesses within a treatment group across the skin locations were mostly non-significant (S6A Fig). The area of the body is only one variable that determines skin thicknesses, others include sex, age, skin tone, weight, and seasons of the year [27–31]. The skin pieces used were from 32–44 age, female donors who had different skin tones, but it was not clear what body weight the donors were or which season of the year the skin was collected. Given that there were only three disparate donors, and they were from different parts of the body, it is not surprising that there were too many variables to tease out significance as compared to the differences seen with the treatment groups.

To better understand which layers BZK enters, it was important to determine the thickness of the epidermis for each treatment group. Measurements were taken of treated skin after being sectioned, sublimated, MALDI imaged, and then stained with hematoxylin and eosin Y (S7 and S9 Figs). Using this staining procedure, the epidermis stained dark purple and the dermis stained pink. Control experiments observed that epidermal thicknesses increased with each step of the procedure (S7B Fig). These results could suggest that the skin went through some degradation and dehydration during the process. This could account for some of the differences in epidermal thicknesses observed between the treatment groups (S7B Fig). Another paper found that there was more water in the stratum corneum of leg as compared to the arm [44]; so perhaps dehydration could have also been one of the reasons that the epidermis thicknesses of arm, leg, and abdomen tissues in our study were not significant within the treatment groups. The size and number of perforations of the dermis also increased after MALDI imaging (S7A Fig). Regardless, significant variations of overall calculated BZK penetration depths were observed between the treatment groups (S8 Fig).

The thickness of the epidermis was found to differ between the treatment groups. When vitamin E was applied to skin, it can become absorbed into different skin layers, and can cause the stratum corneum to become thicker [45, 46]. The thickest average epidermis was observed when skin was treated with the BZK + vitamin E solution. Thus, vitamin E could be additional protection from the dehydration and degradation that occurs throughout the imaging process. Preservative, caprylyl glycol is a surfactant that can disrupt membranes [34, 47]. In our study, treatment with BZK + caprylyl glycol had epidermal thicknesses similar to skin treated with just water. However, Solution 1, which contained caprylyl glycol, had the thinnest epidermis, which contained multiple excipients that were not individually tested. Other additives present in Solution 1 could result in dehydration of the skin resulting in its significantly thinner epidermis.

### BZK penetration

To understand if BZK penetrates skin, we employed MALDI imaging. The USP requires that 70% of the BZK molecules used as antimicrobials be either the C_12_ or C_14_ variant [9]. Of that 70%, at least 40% needs to be C_14_ and at least 20% needs to be C_12_ to meet guidelines. To distribute commercial antiseptics containing BZK, the FDA currently stipulates 0.1% to 0.13% BZK should be used, and our solutions were in this range [1]. The in-house BZK molecules included 70% of C_12_ (*m/z* 304 ion) and 30% of C_14_ (*m/z* 332 ion) alkyl lengths (S2 Table). Although not significant, a trend was noticed that the ion *m/z* 304 typically penetrated deeper into the skin than ion *m/z* 332 (Fig 3). Utilized BZK solutions contained higher concentrations of the C_12_ (ion *m/z* 304) which may account for this observed trend (Fig 3;Table 2; S2 Table).

To better elucidate the influence of other excipients on BZK penetration, several solutions were tested. A significant increase in BZK penetration was observed in the BZK + citric acid solution compared to the positive control which was not pH adjusted (Figs 1, 2 and 4; Table 2). This suggests that pH plays a role in the penetration of BZK. Previous studies have shown that vitamin E and caprylyl glycol on their own can penetrate and localize with the different layers of skin [34, 45]. The inclusion of one of these compounds resulted in a significant decrease in BZK penetration despite having the pH adjusted to 4.7 (Figs 1, 2 and 4; Table 2). Commercial BZK solutions tested were more complex as compared to the in-house prepared solutions. But both commercial BZK solutions contained vitamin E and matched the w/v concentration of the BZK + vitamin E solution. Despite this, both commercial solutions BZK penetrated further into the dermis as compared to the BZK + vitamin E solution (Figs 1, 2 and 4; Table 2). Solution 1 included caprylyl glycol (0.25% w/v) which contained less than the BZK + caprylyl glycol solution (1.0% w/v), which could partially explain the significant increase in BZK penetration observed in Solution 1 compared to the BZK + caprylyl glycol solution (Figs 1, 2 and 4; Table 2). Citric acid was used to acidify both Solution 1 (pH 4.7) and Solution 2 (pH 3.1). Solution 2 had a lower pH than the other tested solutions and an increased penetration compared to both Solution 1 and the positive control (Figs 1, 2 and 4; Table 2). This could highlight the role of pH in influencing the penetration of BZK into skin. However, the BZK + citric acid solution (pH 4.7) by comparison penetrated deeper than Solution 2 (pH 3.1) (Figs 1, 2 and 4; Table 2). Thus, though pH impacts BZK penetration it is not the only factor. This suggests that adding an excipient could result in lower BZK penetration at the same pH. The inclusion of multiple excipients at the same pH, such as with Solution 1, can increase BZK penetration but not match the penetration achieved of just adjusting the pH alone. Overall, the commercial solutions increased penetration of BZK into the layers of the skin compared to the positive control. In the future, other additives, such as sodium benzoate used in Solution 1 and methylchloroisothiazolinone used in Solution 2, could be analyzed for their individual influence on BZK penetration. Similarly, differing combinations of additives and pH levels could be explored. All these factors in solutions could be important to understand the penetration of BZK in human skin.

Overall, this study shows that a 0.123% concentration and a 30 second single application of BZK, BZK readily penetrates through the epidermis and into the dermis. Previous studies looking at BZK penetration concentrations of 0.01% and 0.2% BZK were detected in rabbit eyes [15]. Although the eye BZK study used several months of applications, low levels of BZK were still readily detected at the optic nerve. Other studies analyzing molecule penetration into the skin using passive Franz diffusion cells often use much longer treatment times to ensure penetration and analyzing receptor media. However, in this study BZK rapidly penetrated the skin and was detected in both the epidermal and dermal layers. The BZK did not appear to penetrate through the entire skin which may be seen with longer application times.

### Limitations of the study

This study aimed to know if BZK penetrates different layers of skin, and if additives alter these qualities. It is important to note that the amount of available human skin was limited. Therefore, samples from different skin locations were combined, and arm skin was not tested with all test solutions. However, the differences of epidermal thicknesses between the treatment groups of the different skin locations were found to be non-significant in all cases except the BZK + caprylyl glycol (pH 4.7). Additionally, the quantity of BZK in the skin layers was not analyzed. In the future, more quantification could be done using HPLC with UV-spectroscopy or mass spectrometry analysis on extracts of heat-separated skin layers [17].

## Conclusion

This study analyzed whether BZK can penetrate different skin layers *ex vivo* using a single application of different BZK-containing solutions. Penetration levels were analyzed using MALDI imaging and measured localization depths of the BZK ion *m/z* 304 and ion *m/z* 332 after the skin was embedded in gelatin. Gelatin provided a firm suspension for the skin allowing facile slicing and mounting. Additionally, gelatin did not produce any ions in MALDI imaging, which could interfere with BZK analysis. Thus, gelatin was an effective and affordable medium for analysis of BZK associated ions. This study demonstrates that BZK penetrates not only into the epidermis, but also the dermis. Penetration depth of BZK was influenced by the pH and excipients in the solutions. This study helps to better understand the penetration characteristics of topically applied BZK containing solutions.

## Supporting information

S1 Fig Collected spectra of all matrix, excipients, and solutions. Collected mass spectra of **(A)** CHCA matrix, **(B)** sDHB matrix, **(C)** 0.123% BZK solution, **(D)** citric acid, **(E)** 1.0% caprylyl glycol solution, **(F)** 0.1% vitamin E solution, **(G)** BZK + citric acid treatment solution, **(H)** BZK + caprylyl glycol, **(I)** BZK + vitamin E, **(J)** Solution 1, and **(K)** Solution 2. All were collected in reflectron positive mode with 5,000 laser pulses. Mass range was set to *m/z* 100–600. All solutions were spotted with 40 mg/mL CHCA in acetone as the matrix. Labeled peaks of *m/z* 172, 212, 335, and 379 were all found to be CHCA matrix peaks.
(TIF)

S2 Fig Fragmentation of *m/z* 304.3 and *m/z* 332.33 ions. **(A)** Collected CID spectrum of ion *m/z* 304.3. **(B)** BZK C_12_ structure and CID fragments. **(C)** Collected CID spectrum of ion *m/z* 332.33. **(D)** BZK C_14_ structure and CID fragments.
(TIF)

S3 Fig Comparison of freezing media and gelatin negative controls. Negative control skin embedded in PolyFreeze freezing medium showing **(A)** ion *m/z* 304, **(B)** ion *m/z* 332, and **(C)** overlay of both ions. **(D)** Mass spectra of selected raster spot in epidermis of negative control prepared in PolyFreeze. **(E)** Mass spectra of selected raster spot in dermis of negative control prepared in PolyFreeze. Negative control embedded in gelatin showing the presence of ions of interest **(F)** ion *m/z* 304, **(G)** ion *m/z* 332, and **(H)** the overlay of both within the region of interest. **(I)** Mass spectra of selected raster spot in epidermis of negative control embedded in gelatin. **(J)** Mass spectra of selected raster spot in dermis of negative control embedded in gelatin. All ion heat maps are pulled with background images still present. Selected raster spot mass spectra are noted on ion heat maps with labeled yellow circle.
(TIF)

S4 Fig Comparison of epidermis and dermis of positive and negative controls. **(A)** Ion heat map of negative control skin with both *m/z* 304 and *m/z* 332 selected. **(B)** Mass spectra of selected raster spot from the epidermal layer of the negative control. The labeled masses of *m/z* 211.72, *m/z* 334.67, and *m/z* 378.58 are CHCA matrix peaks **(C)** Mass spectra of selected raster spot from the dermal layer of the negative control. Labeled ion peaks of *m/z* 211.73, *m/z* 334.72, and *m/z* 378.59 are CHCA matrix peaks. **(D)** Ion heat map of the positive control skin with both *m/z* 304 and *m/z* 332 selected. **(E)** Mass spectra of selected raster spot in the epidermal layer of the positive control skin. **(F)** Mass spectra of selected raster spot in the dermal layer where BZK ions were visible in the positive control skin. **(G)** Mass spectra of selected raster spot deeper in the dermal layer of the positive control skin where no BZK ions were visible.
(TIF)

S5 Fig Imaging of gelatin compared to negative and positive control. Ion heat maps of gelatin without skin, negative control, and positive control. **(A-C)** Selected ions of *m/z* 172.06 ±0.43, **(D-F)** ion *m/z* 212.04 ± 0.53, **(G-I)** ion *m/z* 335.14 ± 0.84, **(J-L)** and ion *m/z* 379.13 ±0.95 are all CHCA matrix ions and are represented with purple. **(M-O)** Ions heat maps of ion *m/z* 304.30 ±0.76 (BZK C_12_) in red. **(P-R)** Ion heat maps *m/z* 332.33 ± 0.83 (BZK C_14_) in blue.
(TIF)

S6 Fig Epidermis thickness. (**A**) Epidermis thicknesses observed for each of the different skin types for each of the treatment groups. Significance was determine using multiple paired t-test (**B**) Average epidermis thickness for each of the different treatment groups. Significance was determined with one-way ANOVA. Significance is noted with P<0.05 *, P<0.01 **, P< 0.001 ***, and P<0.0001 ****.
(TIF)

S7 Fig Changes to epidermis control experiment. (**A**) Microscope images of skin samples stained following either mounting, sublimation, or imaging. Each are a representative image from the entire measured skin. Epidermis is present on right side of all images and stained purple, dermis is stained pink. Dark purple patches outside of skin sections is the gelatin embedding medium. **(B)** Graph of epidermis measurements from the skin sections depicted in microscope images. Significance was determined using unpaired t-tests. Significance is noted with P<0.05 *, P<0.01 **, P< 0.001 ***, and P<0.0001 ****.
(TIF)

S8 Fig Total ion penetration into human skin. **(A)** Average penetration observed of ion *m/z* 304 into the skin. **(B)** Average penetration observed of ion *m/z* 332 into the skin. Both are showing the observed thickness of the epidermis in purple and continued ion depth into the dermis in pink.
(TIF)

S9 Fig Comparison of stained skin to BZK ion localization. Ion heat maps containing both *m/z* 304 and *m/z* 332 selected with and without the image background visible. Each is paired with microscope images of the skin following H & E staining. **(A)** Positive control, **(B)** negative control, **(C)** BZK + citric acid (pH 4.7), **(D)** solution 1, **(E)** solution 2, **(F)** BZK + caprylyl glycol, **(G)** BZK + vitamin E.
(TIF)

S1 Table Commercial solution compositions. (XLSX)

S2 Table Benzalkonium chloride alkyl chain length distributions. Reported distribution of alkyl chain lengths present in BZK utilized to make solutions. In-house solutions used Benzalkonium Chloride part #12060 and Rockline Industries used Benzalkonium Chloride, 50%.
(XLSX)

S3 Table Collected mass list of 0.123% BZK. Mass list collected of 0.123% BZK solution spotted with 40 mg/mL CHCA mixed with acetone. Rows highlighted in green correspond to confirmed BZK ions. Rows highlighted in yellow correspond to matrix ions matching those observed in matrix scans.
(XLSX)

S4 Table Epidermal thicknesses of each specific skin location based on treatment group. (XLSX)

S5 Table Average difference in BZK ion penetration. Differences between the average penetration depth of ion *m/z* 304 to ion *m/z* 332. A positive value indicates ion *m/z* 304 was observed deeper while a negative value indicated ion *m/z* 332 penetrated deeper.
(XLSX)

S6 Table Pearson’s correlation coefficient values. Recorded Pearson’s correlation coefficient values for each skin section MALDI imaging analysis based on comparing its *m/z* 304 ion heat map to its *m/z* 332 ion heat map.
(XLSX)
