# Supplementary figures and images for: MALDI-TOF imaging analysis of benzalkonium chloride penetration in ex vivo human skin
Source: PLoS One. 2024 Feb 8;19(2):e0297992. doi: 10.1371/journal.pone.0297992 (PMC10852276; doi:10.1371/journal.pone.0297992)

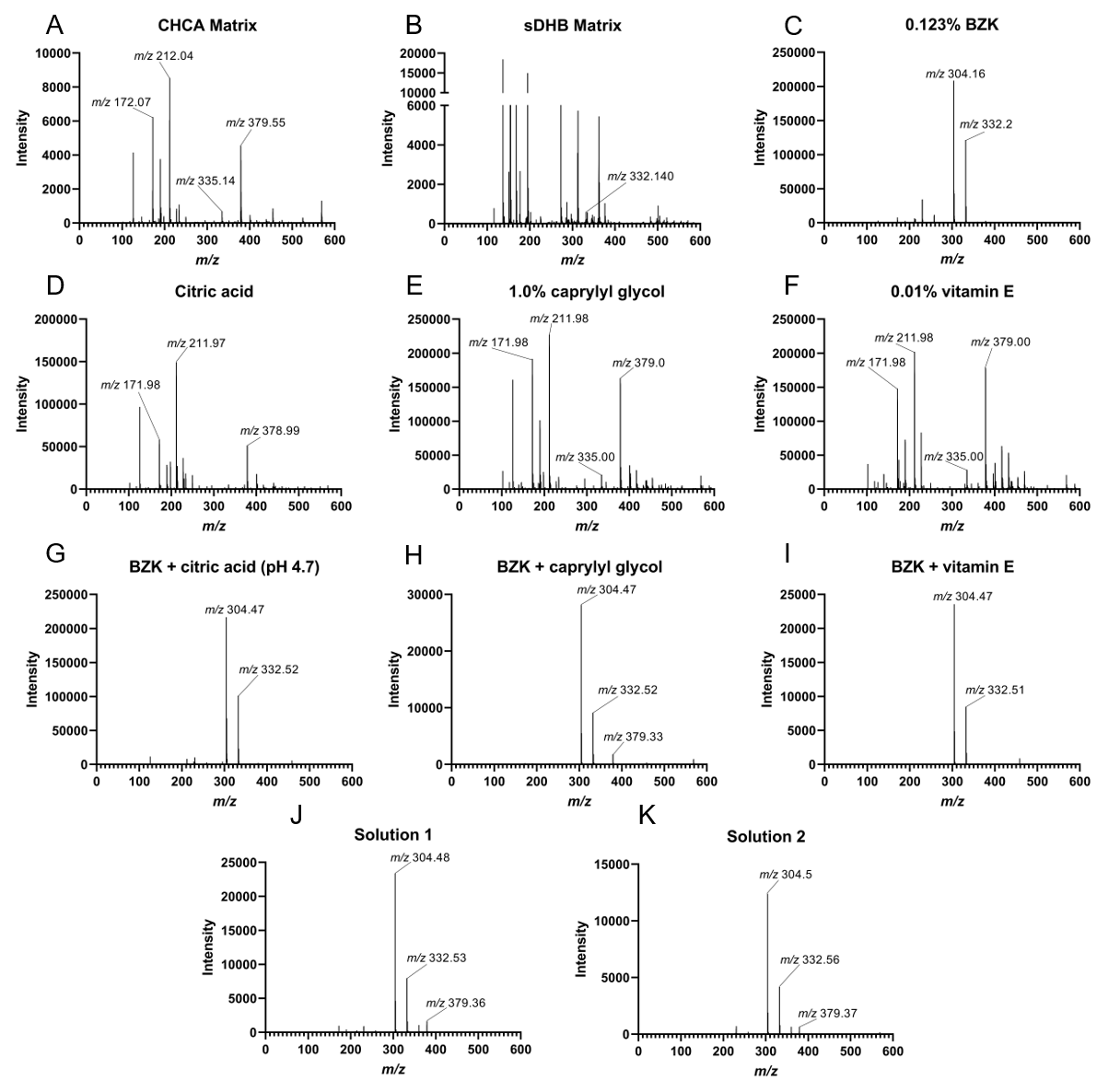

Supplement: S1 Fig — Collected mass spectra of (A) CHCA matrix, (B) sDHB matrix, (C) 0.123% BZK solution, (D) citric acid, (E) 1.0% caprylyl glycol solution, (F) 0.1% vitamin E solution, (G) BZK + citric acid treatment solution, (H) BZK + caprylyl glycol, (I) BZK + vitamin E, (J) Solution 1, and (K) Solution 2. All were collected in reflectron positive mode with 5,000 laser pulses. Mass range was set to m/z 100–600. All solutions were spotted with 40 mg/mL CHCA in acetone as the matrix. Labeled peaks of m/z 172, 212, 335, and 379 were all found to be CHCA matrix peaks. (TIF) [file pone.0297992.s001.tif]

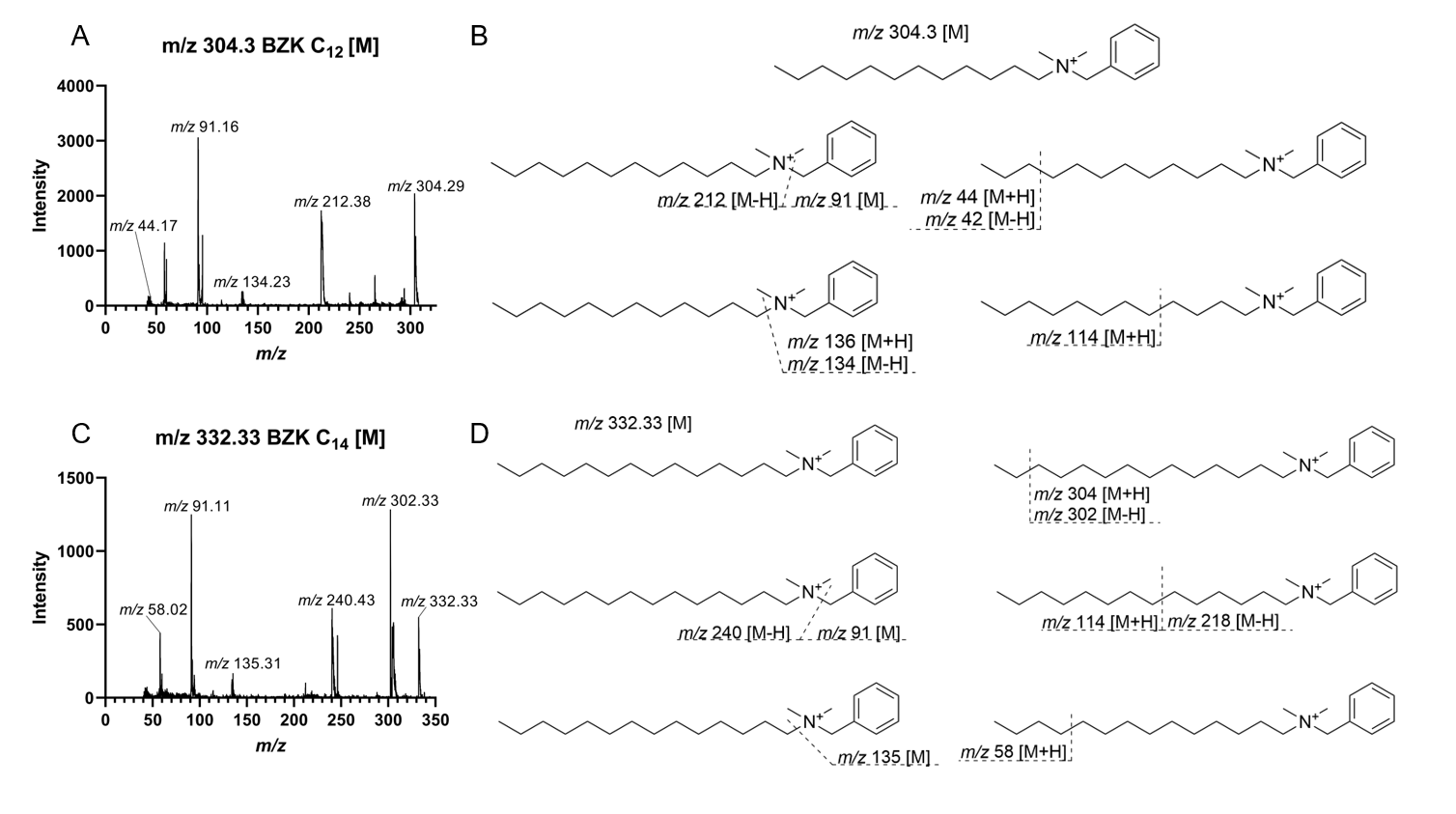

Supplement: S2 Fig — (A) Collected CID spectrum of ion m/z 304.3. (B) BZK C12 structure and CID fragments. (C) Collected CID spectrum of ion m/z 332.33. (D) BZK C14 structure and CID fragments. (TIF) [file pone.0297992.s002.tif]

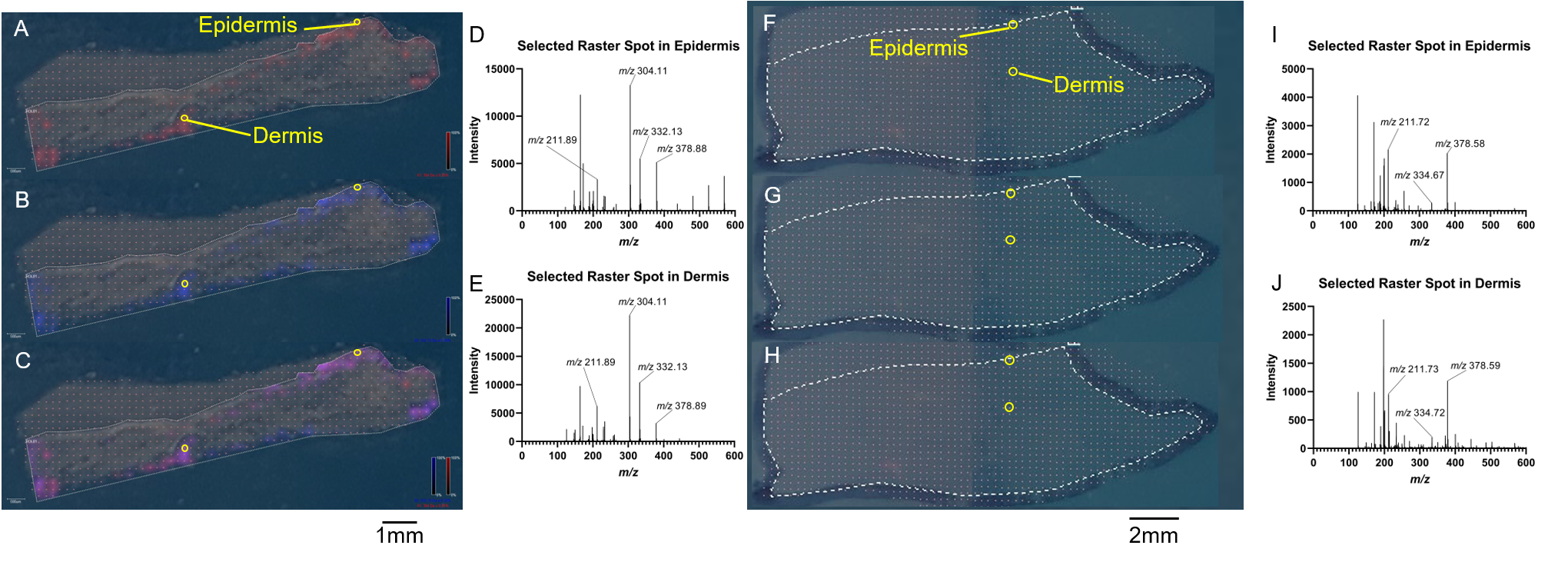

Supplement: S3 Fig — Negative control skin embedded in PolyFreeze freezing medium showing (A) ion m/z 304, (B) ion m/z 332, and (C) overlay of both ions. (D) Mass spectra of selected raster spot in epidermis of negative control prepared in PolyFreeze. (E) Mass spectra of selected raster spot in dermis of negative control prepared in PolyFreeze. Negative control embedded in gelatin showing the presence of ions of interest (F) ion m/z 304, (G) ion m/z 332, and (H) the overlay of both within the region of interest. (I) Mass spectra of selected raster spot in epidermis of negative control embedded in gelatin. (J) Mass spectra of selected raster spot in dermis of negative control embedded in gelatin. All ion heat maps are pulled with background images still present. Selected raster spot mass spectra are noted on ion heat maps with labeled yellow circle. (TIF) [file pone.0297992.s003.tif]

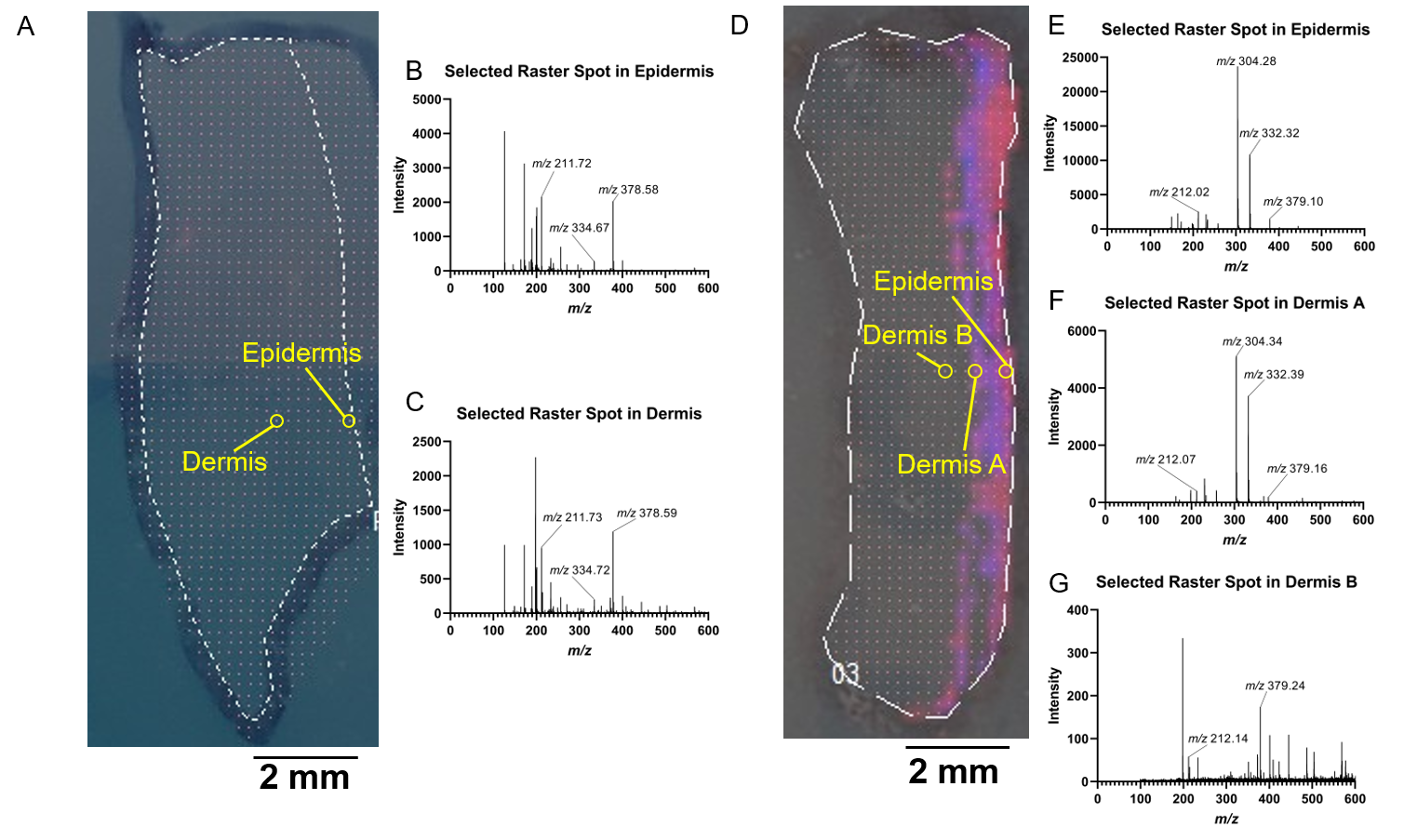

Supplement: S4 Fig — (A) Ion heat map of negative control skin with both m/z 304 and m/z 332 selected. (B) Mass spectra of selected raster spot from the epidermal layer of the negative control. The labeled masses of m/z 211.72, m/z 334.67, and m/z 378.58 are CHCA matrix peaks (C) Mass spectra of selected raster spot from the dermal layer of the negative control. Labeled ion peaks of m/z 211.73, m/z 334.72, and m/z 378.59 are CHCA matrix peaks. (D) Ion heat map of the positive control skin with both m/z 304 and m/z 332 selected. (E) Mass spectra of selected raster spot in the epidermal layer of the positive control skin. (F) Mass spectra of selected raster spot in the dermal layer where BZK ions were visible in the positive control skin. (G) Mass spectra of selected raster spot deeper in the dermal layer of the positive control skin where no BZK ions were visible. (TIF) [file pone.0297992.s004.tif]

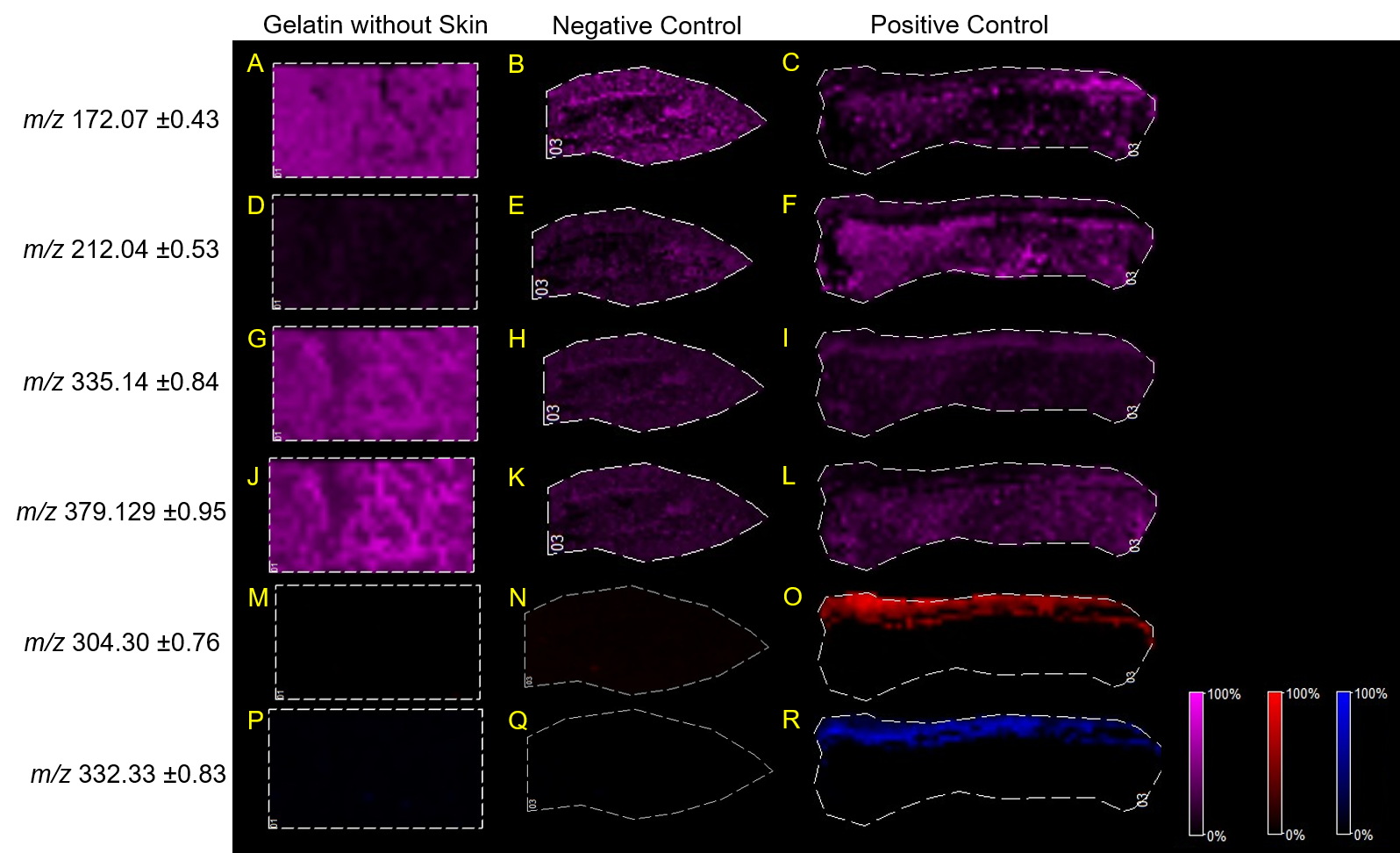

Supplement: S5 Fig — Ion heat maps of gelatin without skin, negative control, and positive control. (A-C) Selected ions of m/z 172.06 ±0.43, (D-F) ion m/z 212.04 ± 0.53, (G-I) ion m/z 335.14 ± 0.84, (J-L) and ion m/z 379.13 ±0.95 are all CHCA matrix ions and are represented with purple. (M-O) Ions heat maps of ion m/z 304.30 ±0.76 (BZK C12) in red. (P-R) Ion heat maps m/z 332.33 ± 0.83 (BZK C14) in blue. (TIF) [file pone.0297992.s005.tif]

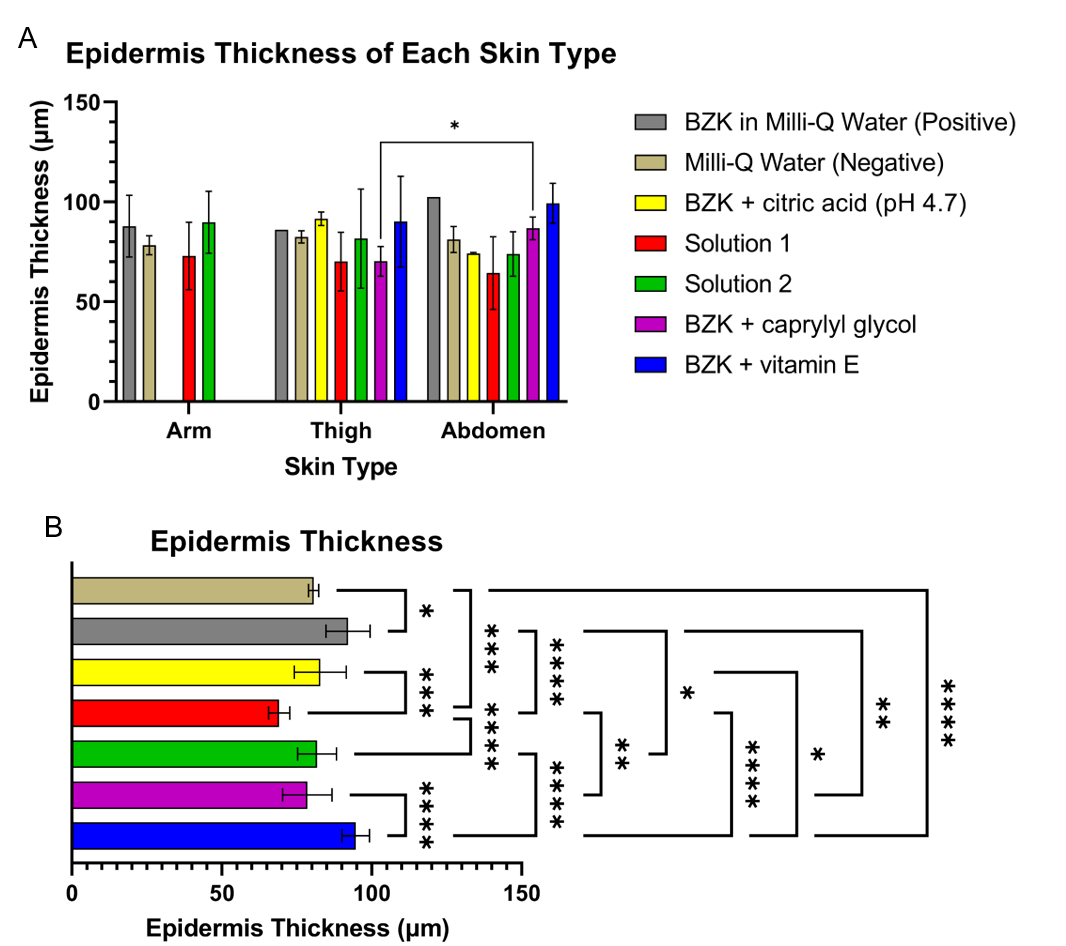

Supplement: S6 Fig — (A) Epidermis thicknesses observed for each of the different skin types for each of the treatment groups. Significance was determine using multiple paired t-test (B) Average epidermis thickness for each of the different treatment groups. Significance was determined with one-way ANOVA. Significance is noted with P<0.05 *, P<0.01 **, P< 0.001 ***, and P<0.0001 ****. (TIF) [file pone.0297992.s006.tif]

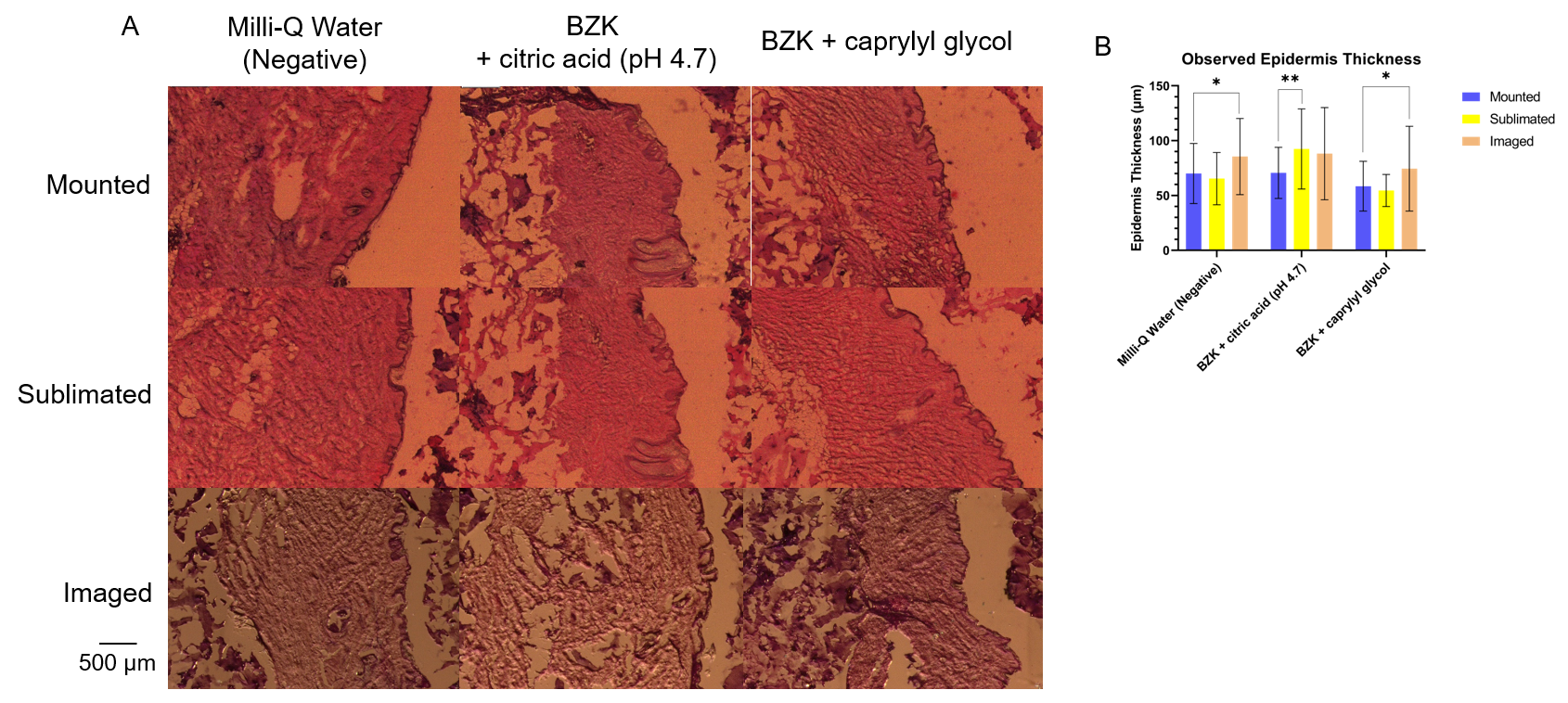

Supplement: S7 Fig — (A) Microscope images of skin samples stained following either mounting, sublimation, or imaging. Each are a representative image from the entire measured skin. Epidermis is present on right side of all images and stained purple, dermis is stained pink. Dark purple patches outside of skin sections is the gelatin embedding medium. (B) Graph of epidermis measurements from the skin sections depicted in microscope images. Significance was determined using unpaired t-tests. Significance is noted with P<0.05 *, P<0.01 **, P< 0.001 ***, and P<0.0001 ****. (TIF) [file pone.0297992.s007.tif]

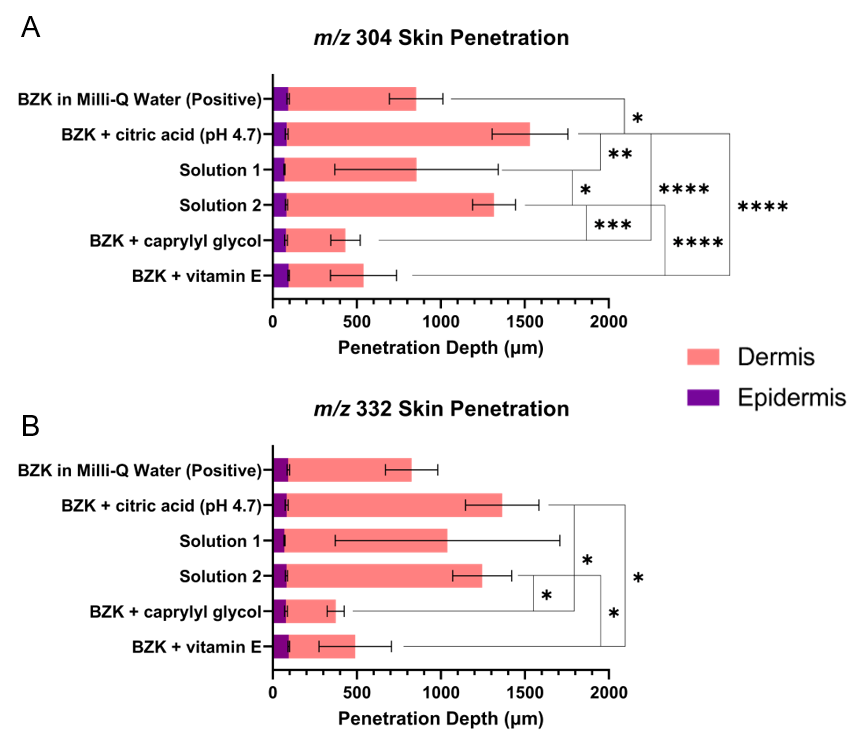

Supplement: S8 Fig — (A) Average penetration observed of ion m/z 304 into the skin. (B) Average penetration observed of ion m/z 332 into the skin. Both are showing the observed thickness of the epidermis in purple and continued ion depth into the dermis in pink. (TIF) [file pone.0297992.s008.tif]

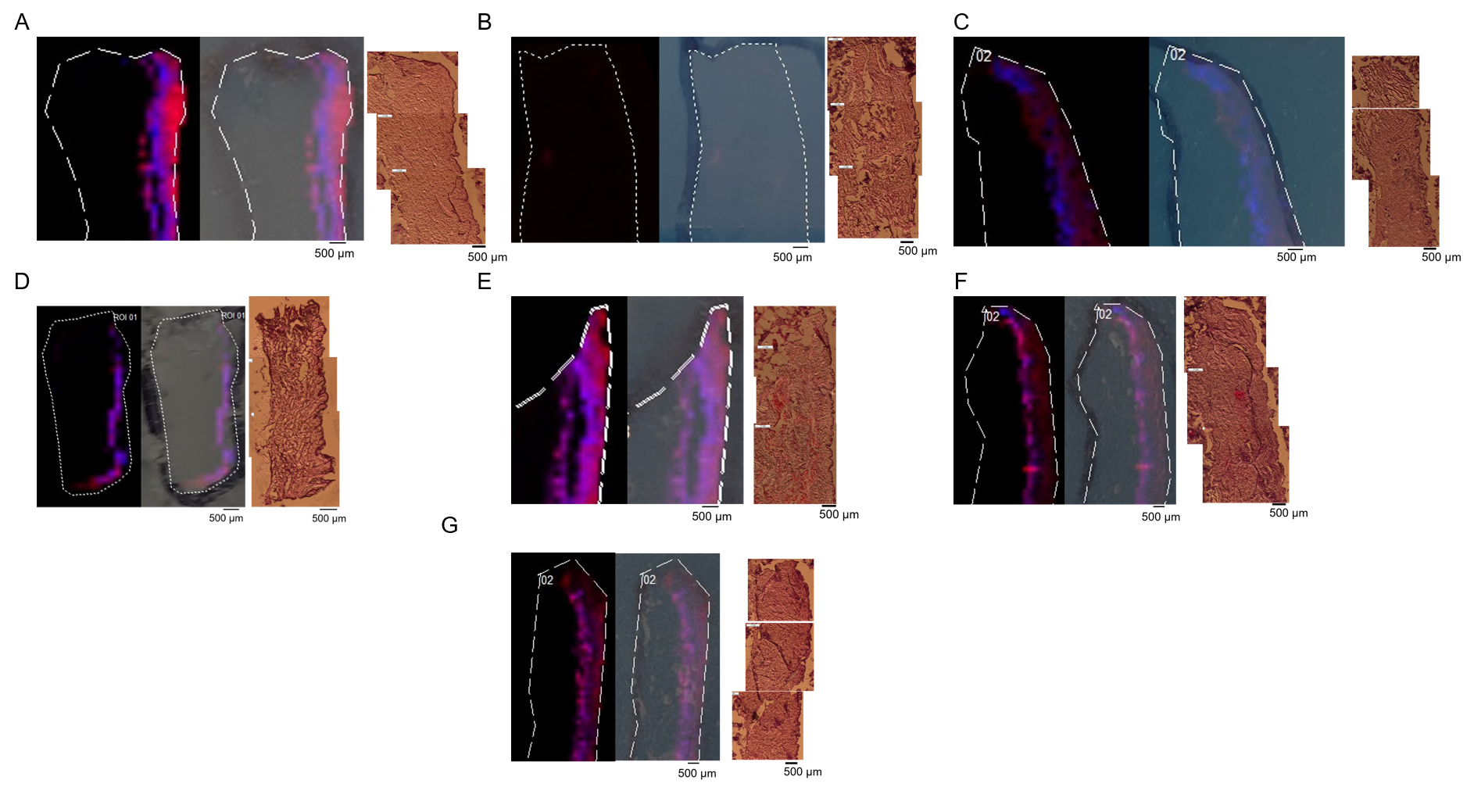

Supplement: S9 Fig — Ion heat maps containing both m/z 304 and m/z 332 selected with and without the image background visible. Each is paired with microscope images of the skin following H & E staining. (A) Positive control, (B) negative control, (C) BZK + citric acid (pH 4.7), (D) solution 1, (E) solution 2, (F) BZK + caprylyl glycol, (G) BZK + vitamin E. (TIF) [file pone.0297992.s009.tif]
